# Seroprevalence of hepatitis E virus antibodies in adults and children from upstate New York: A cross-sectional study

**DOI:** 10.1371/journal.pone.0245850

**Published:** 2021-01-26

**Authors:** Brittany L. Kmush, Amelia M. Lu, Taylor Spillane, Bryce Hruska, Brooks B. Gump, Kestutis G. Bendinskas

**Affiliations:** 1 Department of Public Health, Falk College of Sport and Human Dynamics, Syracuse University, Syracuse, New York, United States of America; 2 Department of Chemistry, State University of New York, Oswego, New York, United States of America; Centers for Disease Control and Prevention, UNITED STATES

## Abstract

Hepatitis E virus (HEV) is a major cause of viral hepatitis around the world, especially in developing countries. Recently, HEV has also been recognized as important cause of hepatitis in Europe and Japan, however, there is a paucity of clinical data from the United States. The overall seroprevalence of HEV antibodies is around 10% in the United States, but considerable variation is seen based on geographic location, year, and assay used. In this study, 63 adults and 417 children from New York State were tested for anti-HEV IgG antibodies using the commercially available Wantai IgG assay. The overall seroprevalence of HEV antibodies among adult participants was 9.52% (95% CI: 3.58–19.59%). Positive adults tended to be older than HEV negative adults, all positive adults were female. Only 3 (0.7%, 95% CI:: 0.15–2.09%) of the children were positive, all positive children were male. These results are consistent with global and United States trends in HEV seroprevalence.

## Introduction

Hepatitis E virus (HEV) is the most common reason for acute viral hepatitis globally causing substantial morbidity and mortality in southeast Asia and sub-Saharan Africa [1]. The virus causes significant complications in pregnant women, with a mortality rate around 20–25%, as well as among individuals with pre-existing liver disease or immunocompromised conditions [2]. What was once a disease of developing countries is now detected in developed countries such as the United States, Europe, and Japan [2]. There are four genotypes of HEV. Genotypes 1 and 2 are prevalent in Southeast Asia, Africa, and South America and typically spread via contaminated water. Genotypes 3 and 4 are found in the United States, Europe, and Japan and often transmitted through ingestion of contaminated meat, usually pork, or transfusions [2].

Few locally acquired clinical cases of HEV have been reported in the United States (US) [3]. However, the true burden of HEV in the US is difficult to measure as HEV is not a nationally notifiable disease and the Food and Drug Administration has not licensed a diagnostic assay. Seroprevalence studies using stored samples as part of the National Health and Nutrition Examination Survey (NHANES) have found relatively high prevalence of past exposure to HEV. These estimates range from 4.5% to 21% in the general US population ≥6 years old, depending on assay used to detect HEV antibodies and year of the study [4–7]. In general, HEV seroprevalence has declined from 1988 to 2016 [4, 7]. Older age is the most consistent risk factor for past exposure to HEV in the United States [4–7]. There is also considerable local variation in antibody prevalence, with the general population in the Mid-Western States tending to have the highest prevalence, possibly due to increased exposure to swine in those areas [8]. However, many studies examining risks for HEV infection at the local, rather than national level, focus on high risk populations (ie: swine veterinarians, acute liver failure patients, or travelers) or blood donors (adults only) rather than members of the general population [9–11].

To overcome the limitations of past research, the current study seeks to clarify the seroprevalence of HEV among a general population sample. Here, we present the results of a cross-sectional seroprevalence study examining a convenience sample of adults and children from New York State for HEV IgG antibodies using the highly sensitive and specific, commercially available Wantai Assay.

## Methods

We conducted a cross-sectional analysis of HEV antibodies in two convenience populations who had stored biological specimen available for analysis. Two groups of participants were used in this study, adults from a study on the physiological effects of taking time off work and children from an environmental toxicant exposure study.

Adult participants from Syracuse, NY were recruited in 2015–2016 for a vocational health study. Adults at least 18 years old in the Syracuse, NY area were eligible to participate if they were employed full-time, were eligible for paid time off, had plans to take a vacation at least 1 month in the future, could read and understand English, and had access to the internet with a working email address. Individuals were excluded if the planned vacations were over Thanksgiving or Christmas. University faculty or schoolteachers were also excluded. Individuals with adrenal gland, pituitary gland, inflammatory/auto-inflammatory disorders, or who were taking medications with long-term effects on the hypothalamic pituitary adrenal axis were also excluded. The sample size and power calculations were made based on the goals of the original study. Medical history, along with race, educational attainment, and other personal information, was self-reported. Additional information about this study can be found elsewhere [12, 13]. Sixty-seven adults met the inclusion and exclusion criteria, of whom 63 agreed to participate (4 declined to participate due to insufficient time). The 63 individuals included in the original study, were included in this analysis.

Children were recruited from the Environmental Exposures and Child Health Outcomes (EECHO) study examining the association between environmental toxicants and cardiovascular risk indices. The EECHO study aimed to recruit approximately equal numbers of low-income African American and Caucasian children from the Syracuse, NY area. Children that did not self-identify as either African American or Caucasian, were not 9–11 years old, did not meet the zip code residence selection criteria (designed to target low SES neighborhoods), those with serious medical or developmental disabilities, and those who were on medications that might affect their cardiovascular health were excluded. However, a few children were later found to be outside of the targeted age range, but still included in the study. The sample size and power calculations were made based on the goals of the original study. Additional information about this study can be found elsewhere [14]. From 2006 to 2007, a pilot study recruited 150 children, of which 127 had demographic information recorded and a sufficient blood sample to complete the HEV analysis. In 2017, 558 children were recruited for the main EECHO study, of whom 290 met the inclusion criteria for the original study and had a sufficient blood draw for HEV analysis. Therefore, 417 children with sufficient demographic information and a blood draw were included in this analysis.

A venous blood draw was collected from all participants. All study procedures were reviewed and approved by the Syracuse University Institutional Review Board. All participants, or their parent or legal guardian where applicable, gave informed written consent as appropriate. Children also gave written assent.

In 2018, antibody testing for HEV immunoglobulin G (IgG) was completed using a commercially available enzyme-linked immunosorbent (ELISA) assay by Beijing Wantai Pharmacy Enterprise Co., Ltd. (Beijing, China) according to the manufacturer’s instructions. This testing was completed at the laboratory of the Department of Chemistry of the State University of New York College at Oswego. The Wantai assay is considered one of the most sensitive and specific assays to detect HEV antibodies [15]. Seroprevalence estimates and exact binomial confidence intervals were calculated separately for the adults and children. Student’s t-tests and χ^2^ tests were used to compare continuous and categorical variables, respectively, in HEV IgG positive and negative participants.

## Results

The anti-HEV IgG prevalence of our adult population was 9.52% (95% CI: 3.58–19.59%), with 6 out of 63 participants testing positive for HEV antibodies. Only 0.7% (95% CI: 0.15–2.09%) of the pediatric participants were positive, 3 out of 417. Out of the 6 positive adult participants, all were female and 5 were college educated. Among the adults, the average age of the positive individuals was 50.8 years, whereas the average of the negative individuals was 42.6 years; this was not a statistically significant difference (p = 0.065) (Table 1). Contrary to the adult population, all 3 of the positive children were male and tended to be younger (8.6 versus 10.0 years, p = 0.011) (Table 1). However, the small number of positive pediatric participants makes it difficult to compare characteristics.

**Table 1:** Participant characteristics from Syracuse, New York (2006–2017).

Study Population	Characteristic	Sero-Positive	Sero-Negative	P-value
Adults (n = 63)		N = 6	N = 57	
	Age (years) (Range)	50.8 (41–60)	42.6 (24–62)	0.065*
	Gender (n(%))			0.09**
	Male	0 (0%)	19 (33.3%)	
	Female	6 (100%)	38 (66.7%)	
	Race (n(%))			0.28**
	African American, Hispanic, and other	1 (16.7%)	3 (5.3%)	
	White	5 (83.3%)	54 (94.7%)	
	Education			0.55**
	Did not earn a college degree	1 (16.7%)	16 (28.1%)	
	College graduate	5 (83.3%)	41 (71.9%)	
Children (n = 417)		N = 3	N = 414	
	Age (years) (Range)	8.6 (6.7–10.0)	10.0 (7.0–15.9)	0.011*
	Gender (n(%))			0.11**
	Male	3 (100%)	222 (53.6%)	
	Female	0 (0%)	192 (46.4%)	

*t-test.

**chi-square test.

Interestingly, two of the adult HEV IgG positive participants presented with a past medical history of hypothyroidism. To our knowledge, there have been published studies regarding the correlation of hepatitis E virus as putative trigger of autoimmune thyroiditis and hyperthyroidism, however, hypothyroidism have not been studied [16]. Additional risk factors for past HEV exposure could not be well assessed using these convenience samples and therefore should be the focus of future studies.

## Discussion

This is the first study to explore the seroprevalence of HEV in both adults and children in the Syracuse, NY area. The seroprevalence in adults found here was 9.52%, which is similar to the United States HEV seroprevalence (8.1%) found in a recent analysis of NHANES [6]. Worldwide, the seroprevalence for children up to the age of 10 is less than 10% [17]. Our results also show a very low seroprevalence in children. In endemic countries, fecal-oral transmission is the primary route for exposure to HEV. In non-endemic countries, like the United States, zoonotic exposure is widely hypothesized to be a potential transmission route in these sporadic and autochthonous HEV infections.

However, it is important to consider that two convenience samples were used in this analysis. The adult participants were employed full-time with paid vacation time, likely representative of a higher socio-economic status than the general population. Children from lower income neighborhoods were targeted for inclusion into this sample. Lower socio-economic status has been associated with the increased risk HEV exposure in South East Asia [18]. However, the role of socio-economic status in HEV infection is not well established in developed countries and needs further examination.

It is difficult to pinpoint exact routes for transmission for HEV infections in developed countries such as the United States since the manifestation of disease, source of infection, and route of transmission remains relatively ambiguous. We speculate that zoonotic factors such as proximity or direct contact with an infected animal source along with foodborne and environmental exposures may play a role in HEV acquisition for our study population. Close contact with swine or consumption of under-cooked pork may be a significant mechanism of acquiring HEV in developed countries [17]. Furthermore, serological evidence of HEV has been found in cattle, sheep, rabbits, mongooses, chickens, goats, cats, dogs, and rhesus monkeys, although the virus has not been isolated or sequenced from all of these animals [19].

Furthermore, ingesting self-grown food has been hypothesized to be another risk factor for seropositivity in the United States [20]. Dietary intake may play a role in the causal pathway to HEV infection in this study. Unfortunately, dietary data was only assessed in a sub-set of our participants, including only one seropositive child. Therefore, future studies should aim to correlate the relationship between dietary intake in Upstate New York and its role in HEV serology.

In upstate New York, a large portion of the state is rural farmland where zoonotic exposure is a high possibility along with other risk factors such as ingesting self-grown food, and consumption of raw or undercook animal products. Further studies should be conducted to assess zoonotic exposures and ingestion of self-grown food as a possible risk factors for HEV in countries like the United States.

## Supporting information

S1 Dataset Supporting data. (XLSX)
